# A New Method of Preserving the Dead

**Published:** 1867-09

**Authors:** 


A NEW METHOD OF PRESERVING THE DEAD.—There is now on exhibition at the New York Morgue, the body of a drowned man, supposed to have been in the water for three days prior to its recovery, and which is being subjected to an experimental preservation. It is inclosed in a metallic case, made perfectly air-tight, and, as yet, although forty days have elapsed since the commencement of the experiment, shows no signs of decomposition. This result is obtained by forcing the air from the case and supplying its place with a certain gas, which the discoverer, we regret to state, is inclined to keep secret. He even expresses the belief that the body in course of time will become as hard as stone.—*Medical Record.*

